# Supplementary material for: Interplay of two small RNAs fine-tunes hierarchical flagella gene expression in Campylobacter jejuni
Source: Nat Commun. 2024 Jun 19;15:5240. doi: 10.1038/s41467-024-48986-8 (PMC11187230; doi:10.1038/s41467-024-48986-8)
Supplement: Supplementary file 1 — Supplementary Information [file 41467_2024_48986_MOESM1_ESM.pdf]

Supplementary Information for

Interplay of two small RNAs fine-tunes hierarchical flagella gene expression in *Campylobacter jejuni*

Fabian König, Sarah L. Svensson and Cynthia M. Sharma

Corresponding author: Cynthia M. Sharma

Email: cynthia.sharma@uni-wuerzburg.de

This PDF file includes:

Supplementary Figures 1-12

Supplementary References

Supplementary Figures

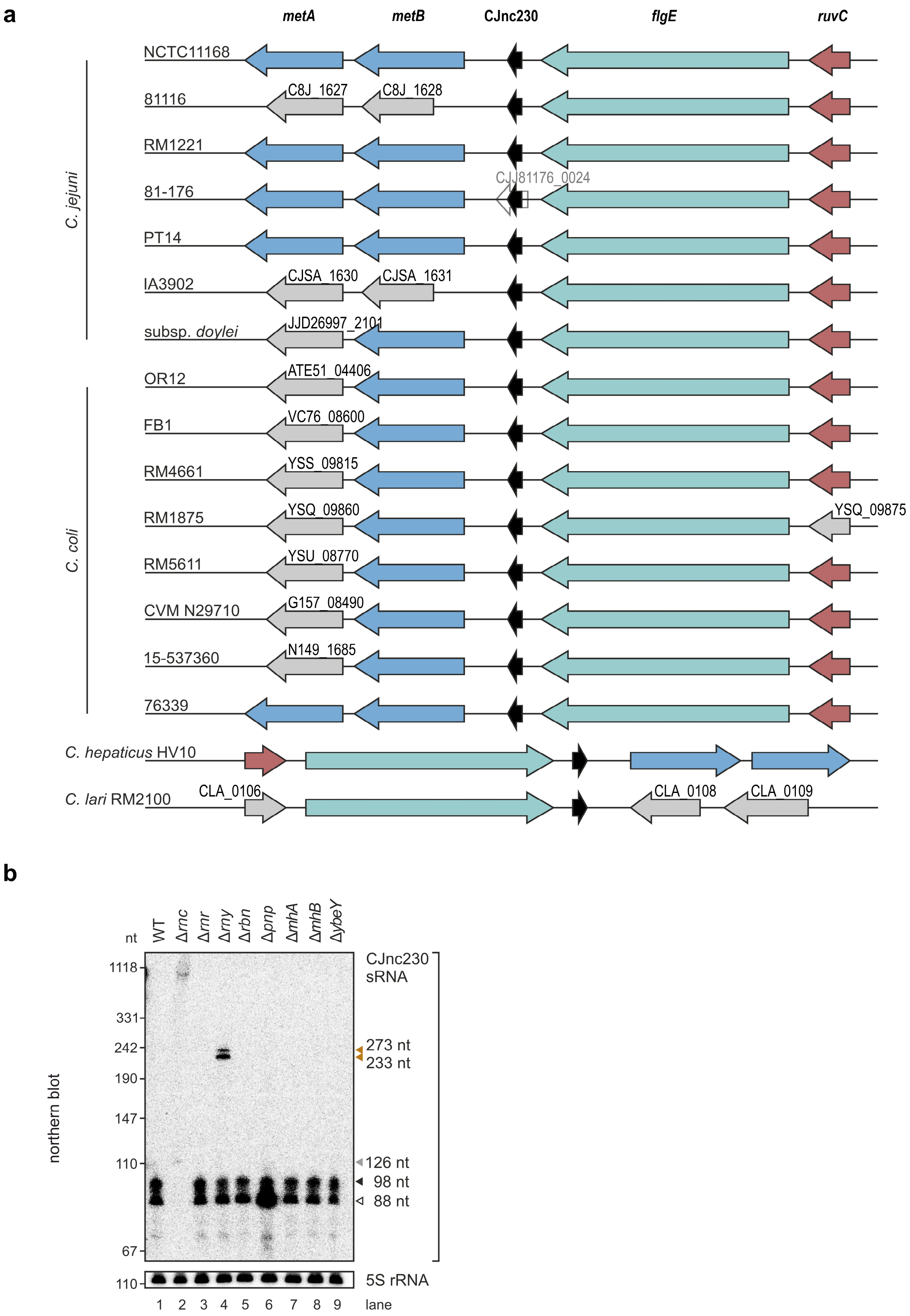

Supplementary Figure 1. CJnc230 sRNA encoded downstream of the *flgE* gene is processed by several ribonucleases. **a**, Conservation analysis of the CJnc230 genomic locus in *Campylobacter* species and strains used for sequence alignments in **Figure 1c and Supplementary Figure 4**. Information was retrieved from KEGG (Kyoto Encyclopedia of Genes and Genomes) and orthologous genes from different strains and species are color-coded: *ruvC* - red, *flgE* (Cj1729c in NCTC11168) - turquoise, CJnc230 - black, *metB/A* - blue, hypothetical and other function - gray. For *C. jejuni* strain 81-176, a small open reading frame (CJJ81176_0024, 51 aa) is predicted to overlap with CJnc230. Sizes of arrows, indicating genes and their orientation, are not displayed at the correct scale. **b**, Northern blot analysis of total RNA from *C. jejuni* wildtype (WT) and ribonuclease deletion strains grown to exponential phase. Transcript lengths were calculated based on 5' and 3' end positions determined by primer extension or term-seq, respectively, and marked by colored triangles (white: 88 nt, 3'-truncated version of CJnc230; black: 98 nt, full-length version of CJnc230; gray: 126 nt, possibly 5'-extended CJnc230 detected in Δ*rnc*; orange: 233/273 nt, 3'-extended versions of CJnc230 detected in Δ*rny*). Expression of CJnc230 sRNA was detected with CSO-0537 and 5S rRNA (CSO-0192) served as a loading control. Related to **Figure 2**. Data in (**b**) are representative results of at least two independent experiments. Source data are provided as a Source Data File.

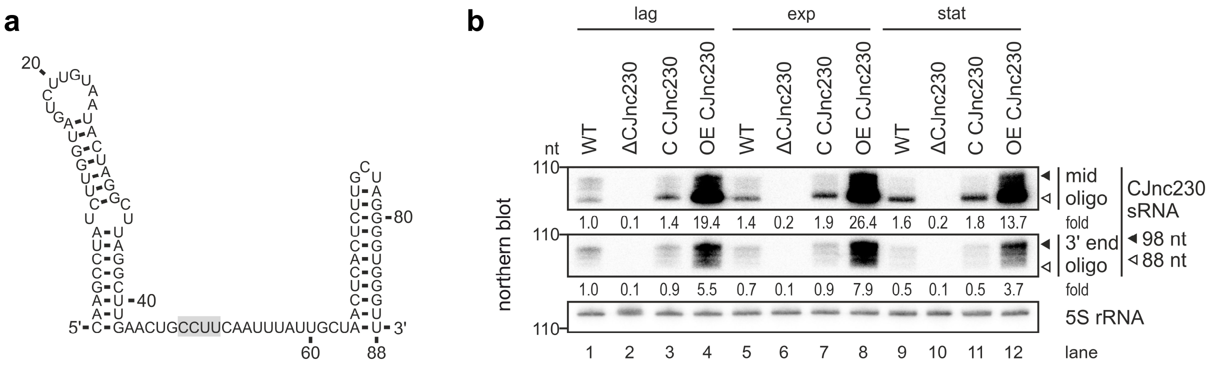

c

cRACE CJnc230 WT

```
                                        mid oligo                          3' end oligo
 98 nt  5'-CAAGCCTATCTTGGTAGTCTTGTAATACTAGGCTTAGGCTTGAACTGCCTTCAATTTATTGCTAACTCACTCTTGCTAGGGGTGGGTTGCAATAAAAT-3'
 97 nt  5'-CAAGCCTATCTTGGTAGTCTTGTAATACTAGGCTTAGGCTTGAACTGCCTTCAATTTATTGCTAACTCACTCTTGCTAGGGGTGGGTTGCAATAAAA.-3'
 96 nt  5'-CAAGCCTATCTTGGTAGTCTTGTAATACTAGGCTTAGGCTTGAACTGCCTTCAATTTATTGCTAACTCACTCTTGCTAGGGGTGGGTTGCAATAAA..-3'
 96 nt  5'-CAAGCCTATCTTGGTAGTCTTGTAATACTAGGCTTAGGCTTGAACTGCCTTCAATTTATTGCTAACTCACTCTTGCTAGGGGTGGGTTGCAATAAA..-3'
 96 nt  5'-CAAGCCTATCTTGGTAGTCTTGTAATACTAGGCTTAGGCTTGAACTGCCTTCAATTTATTGCTAACTCACTCTTGCTAGGGGTGGGTTGCAATAAA..-3'
 96 nt  5'-CAAGCCTATCTTGGTAGTCTTGTAATACTAGGCTTAGGCTTGAACTGCCTTCAATTTATTGCTAACTCACTCTTGCTAGGGGTGGGTTGCAATAAA..-3'
 94 nt  5'-CAAGCCTATCTTGGTAGTCTTGTAATACTAGGCTTAGGCTTGAACTGCCTTCAATTTATTGCTAACTCACTCTTGCTAGGGGTGGGTTGCAATA....-3'
 94 nt  5'-CAAGCCTATCTTGGTAGTCTTGTAATACTAGGCTTAGGCTTGAACTGCCTTCAATTTATTGCTAACTCACTCTTGCTAGGGGTGGGTTGCAATA....-3'
 94 nt  5'-CAAGCCTATCTTGGTAGTCTTGTAATACTAGGCTTAGGCTTGAACTGCCTTCAATTTATTGCTAACTCACTCTTGCTAGGGGTGGGTTGCAATA....-3'
 94 nt  5'-CAAGCCTATCTTGGTAGTCTTGTAATACTAGGCTTAGGCTTGAACTGCCTTCAATTTATTGCTAACTCACTCTTGCTAGGGGTGGGTTGCAATA....-3'
 94 nt  5'-CAAGCCTATCTTGGTAGTCTTGTAATACTAGGCTTAGGCTTGAACTGCCTTCAATTTATTGCTAACTCACTCTTGCTAGGGGTGGGTTGCAATA....-3'
 94 nt  5'-CAAGCCTATCTTGGTAGTCTTGTAATACTAGGCTTAGGCTTGAACTGCCTTCAATTTATTGCTAACTCACTCTTGCTAGGGGTGGGTTGCAATA....-3'
 94 nt  5'-CAAGCCTATCTTGGTAGTCTTGTAATACTAGGCTTAGGCTTGAACTGCCTTCAATTTATTGCTAACTCACTCTTGCTAGGGGTGGGTTGCAATA....-3'
 89 nt  5'-CAAGCCTATCTTGGTAGTCTTGTAATACTAGGCTTAGGCTTGAACTGCCTTCAATTTATTGCTAACTCACTCTTGCTAGGGGTGGGTTG.........-3'
 89 nt  5'-CAAGCCTATCTTGGTAGTCTTGTAATACTAGGCTTAGGCTTGAACTGCCTTCAATTTATTGCTAACTCACTCTTGCTAGGGGTGGGTTG.........-3'
 88 nt  5'-....CCTATCTTGGTAGTCTTGTAATACTAGGCTTAGGCTTGAACTGCCTTCAATTTATTGCTAACTCACTCTTGCTAGGGGTGGGTT..........-3'
```

cRACE CJnc230 Δ*rny*

```
106 nt  5'-CAAGCCTATCTTGGTAGTCTTGTAATACTAGGCTTAGGCTTGAACTGCCTTCAATTTATTGCTAACTCACTCTTGCTAGGGGTGGGTTGCAATAAAATTTTCAGCA-3'
 97 nt  5'-CAAGCCTATCTTGGTAGTCTTGTAATACTAGGCTTAGGCTTGAACTGCCTTCAATTTATTGCTAACTCACTCTTGCTAGGGGTGGGTTGCAATAAAA.-3'
 97 nt  5'-CAAGCCTATCTTGGTAGTCTTGTAATACTAGGCTTAGGCTTGAACTGCCTTCAATTTATTGCTAACTCACTCTTGCTAGGGGTGGGTTGCAATAAAA.-3'
 97 nt  5'-CAAGCCTATCTTGGTAGTCTTGTAATACTAGGCTTAGGCTTGAACTGCCTTCAATTTATTGCTAACTCACTCTTGCTAGGGGTGGGTTGCAATAAAA.-3'
 97 nt  5'-CAAGCCTATCTTGGTAGTCTTGTAATACTAGGCTTAGGCTTGAACTGCCTTCAATTTATTGCTAACTCACTCTTGCTAGGGGTGGGTTGCAATAAAA.-3'
 97 nt  5'-CAAGCCTATCTTGGTAGTCTTGTAATACTAGGCTTAGGCTTGAACTGCCTTCAATTTATTGCTAACTCACTCTTGCTAGGGGTGGGTTGCAATAAAA.-3'
 96 nt  5'-CAAGCCTATCTTGGTAGTCTTGTAATACTAGGCTTAGGCTTGAACTGCCTTCAATTTATTGCTAACTCACTCTTGCTAGGGGTGGGTTGCAATAAA..-3'
 96 nt  5'-CAAGCCTATCTTGGTAGTCTTGTAATACTAGGCTTAGGCTTGAACTGCCTTCAATTTATTGCTAACTCACTCTTGCTAGGGGTGGGTTGCAATAAA..-3'
 96 nt  5'-CAAGCCTATCTTGGTAGTCTTGTAATACTAGGCTTAGGCTTGAACTGCCTTCAATTTATTGCTAACTCACTCTTGCTAGGGGTGGGTTGCAATAAA..-3'
 96 nt  5'-CAAGCCTATCTTGGTAGTCTTGTAATACTAGGCTTAGGCTTGAACTGCCTTCAATTTATTGCTAACTCACTCTTGCTAGGGGTGGGTTGCAATAAA..-3'
 96 nt  5'-CAAGCCTATCTTGGTAGTCTTGTAATACTAGGCTTAGGCTTGAACTGCCTTCAATTTATTGCTAACTCACTCTTGCTAGGGGTGGGTTGCAATAAA..-3'
 96 nt  5'-CAAGCCTATCTTGGTAGTCTTGTAATACTAGGCTTAGGCTTGAACTGCCTTCAATTTATTGCTAACTCACTCTTGCTAGGGGTGGGTTGCAATAAA..-3'
 96 nt  5'-CAAGCCTATCTTGGTAGTCTTGTAATACTAGGCTTAGGCTTGAACTGCCTTCAATTTATTGCTAACTCACTCTTGCTAGGGGTGGGTTGCAATAAA..-3'
 96 nt  5'-CAAGCCTATCTTGGTAGTCTTGTAATACTAGGCTTAGGCTTGAACTGCCTTCAATTTATTGCTAACTCACTCTTGCTAGGGGTGGGTTGCAATAAA..-3'
 96 nt  5'-CAAGCCTATCTTGGTAGTCTTGTAATACTAGGCTTAGGCTTGAACTGCCTTCAATTTATTGCTAACTCACTCTTGCTAGGGGTGGGTTGCAATAAA..-3'
 96 nt  5'-CAAGCCTATCTTGGTAGTCTTGTAATACTAGGCTTAGGCTTGAACTGCCTTCAATTTATTGCTAACTCACTCTTGCTAGGGGTGGGTTGCAATAAA..-3'
 95 nt  5'-CAAGCCTATCTTGGTAGTCTTGTAATACTAGGCTTAGGCTTGAACTGCCTTCAATTTATTGCTAACTCACTCTTGCTAGGGGTGGGTTGCAATAA...-3'
 94 nt  5'-CAAGCCTATCTTGGTAGTCTTGTAATACTAGGCTTAGGCTTGAACTGCCTTCAATTTATTGCTAACTCACTCTTGCTAGGGGTGGGTTGCAATA....-3'
 94 nt  5'-CAAGCCTATCTTGGTAGTCTTGTAATACTAGGCTTAGGCTTGAACTGCCTTCAATTTATTGCTAACTCACTCTTGCTAGGGGTGGGTTGCAATA....-3'
 94 nt  5'-CAAGCCTATCTTGGTAGTCTTGTAATACTAGGCTTAGGCTTGAACTGCCTTCAATTTATTGCTAACTCACTCTTGCTAGGGGTGGGTTGCAATA....-3'
 94 nt  5'-CAAGCCTATCTTGGTAGTCTTGTAATACTAGGCTTAGGCTTGAACTGCCTTCAATTTATTGCTAACTCACTCTTGCTAGGGGTGGGTTGCAATA....-3'
```

Supplementary Figure 2. The 3'-truncated version of CJnc230 increases over growth. **a**, Secondary structure of the most abundant, 3'-truncated 88-nt version of CJnc230 predicted by RNAfold[1]. Gray box: putative anti-Shine-Dalgarno motif. **b**, Northern blot analysis of total RNA from *C. jejuni* WT and CJnc230 mutants (Δ: deletion, C: complementation in *trans*, and OE: overexpression in *trans*) harvested at lag phase (lag, $OD_{600\ nm}$ 0.1), exponential phase (exp, $OD_{600\ nm}$ ~0.4), and stationary phase (stat, $OD_{600\ nm}$ ~0.8). The full-length (black triangle, 98 nt) and the most abundant 3'-truncated version (white triangle, 88 nt) of CJnc230 are marked. CSO-0537 is complementary to the middle part of the sRNA (mid oligo), while CSO-5138 is directed towards the 3' end (3' end oligo) (positions indicated in (**c**)). 5S rRNA (CSO-0192) was used as a loading control. Fold changes of CJnc230 expression relative to WT and normalized to 5S rRNA are indicated. **c**, Sequencing results of circular Rapid Amplification of cDNA End (cRACE) analysis of total RNA from *C. jejuni* WT and Δ*rny* strains harvested at exponential growth phase. Depicted are CJnc230 DNA sequences with respective lengths on the left and oligonucleotide binding positions

for northern blot analysis in (**b**) indicated on top. Data in (**b, c**) are representative results of at least two independent experiments. Source data are provided as a Source Data File.

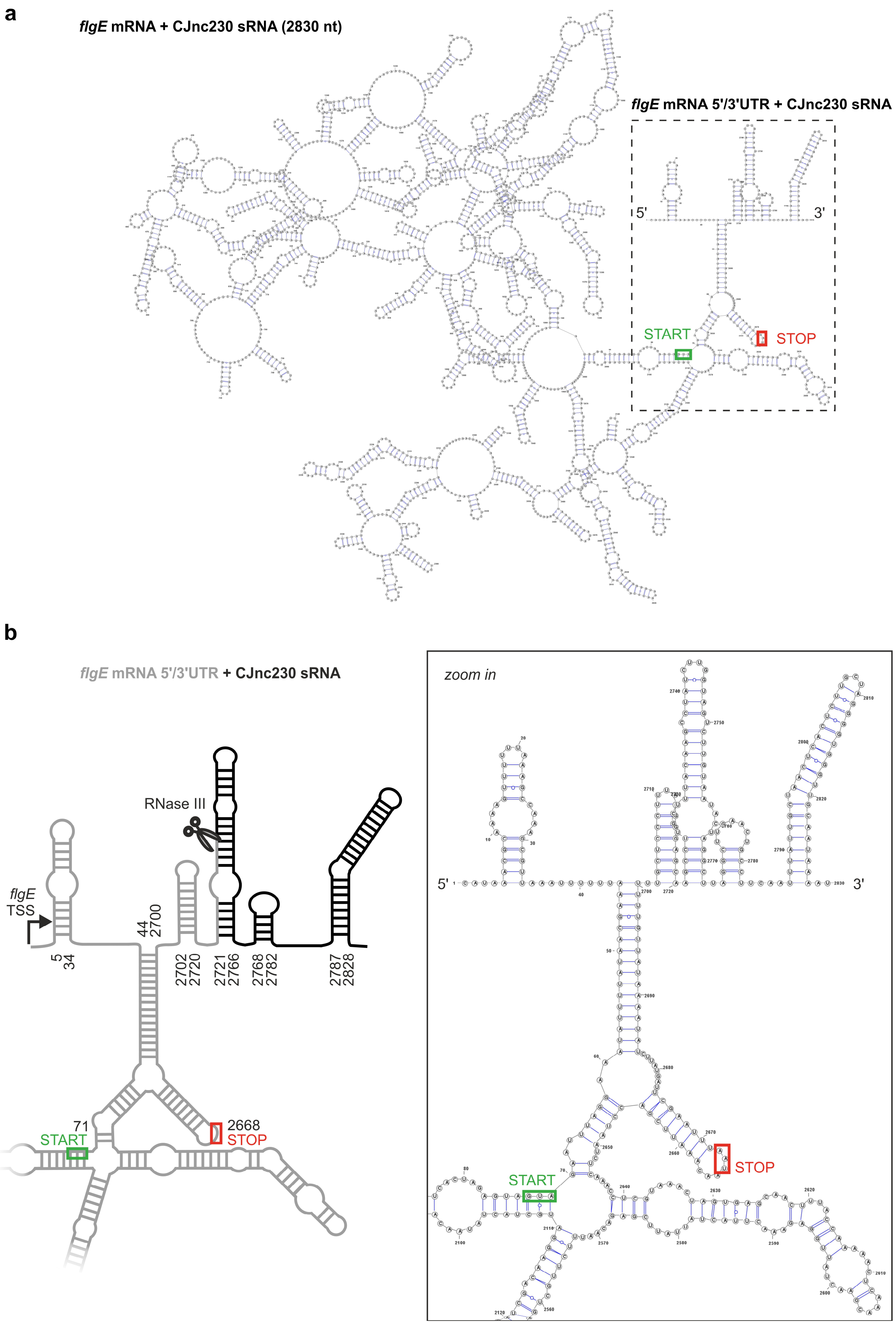
a
flgE mRNA + CJnc230 sRNA (2830 nt)
flgE mRNA 5'/3'UTR + CJnc230 sRNA
5'
3'
START
STOP
b
flgE mRNA 5'/3'UTR + CJnc230 sRNA
RNase III
flgE
TSS
5
34
44
2700
2702
2720
2721
2766
2768
2782
2787
2828
71
START
2668
STOP
zoom in
5'
3'
START
STOP

Supplementary Figure 3. Secondary structure prediction reveals a stem-loop for possible RNase III cleavage downstream of the *flgE* CDS. a, RNA secondary structure prediction of the 2,830-nt long *flgE*-CJnc230 transcript of *C. jejuni* NCTC11168 by RNAfold[1]. The dashed box highlights the *flgE* (Cj1729c) start (green box) and stop (red box) codon, 5'UTR and 3'UTR including the CJnc230 sRNA, and transcript 5' and 3' ends illustrated in more detail in (**b**). **b**, Scheme (*left*) and corresponding zoom in region (*right*) of potential RNase III cleavage (scissors) in a stem-loop downstream of the *flgE* CDS, thus producing the CJnc230 sRNA (black) 5' end. Bent arrow: transcriptional start site (TSS) of the *flgE* mRNA[2]. Stem-loop positions with respect to the TSS are provided in the scheme to estimate sizes. Start (green box) and stop (red box) codon positions of the *flgE* open reading frame are also indicated.

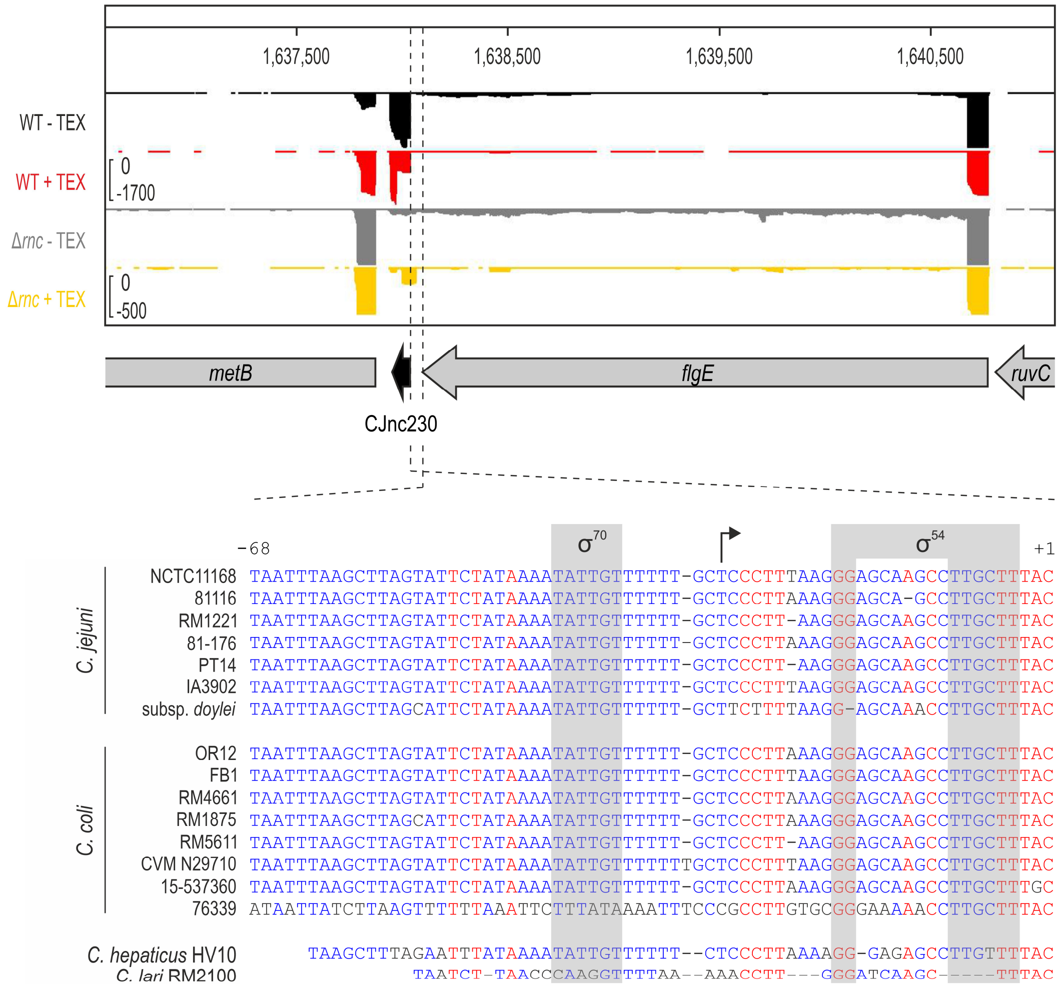

Supplementary Figure 4. Differential RNA sequencing (dRNA-seq) reveals an alternative TSS upstream of CJnc230. (*Upper*) dRNA-seq coverage of the *flgE*-CJnc230 locus in *C. jejuni* WT and the Δ*rnc* mutant grown to exponential phase. -/+TEX: mock-/terminator exonuclease (TEX)-treated dRNA-seq[3] libraries. TEX enriches for 5'-triphosphorylated primary transcript ends, as a consequence of degrading processed (non-triphosphorylated) 5' ends. (*Lower*) Genomic sequence alignment by MultAlin[4] of multiple *Campylobacter* species and strains comprising the *flgE* 3'UTR from the stop codon (TAA, positions -68 to -66) to the CJnc230 5' end (processing site, C residue, +1). Gray boxes: two promoter motifs for σ^{70} and σ^{54}, respectively. Bent arrow: TSS detected by dRNA-seq and primer extension (**Fig. 2b**) in Δ*rnc*.

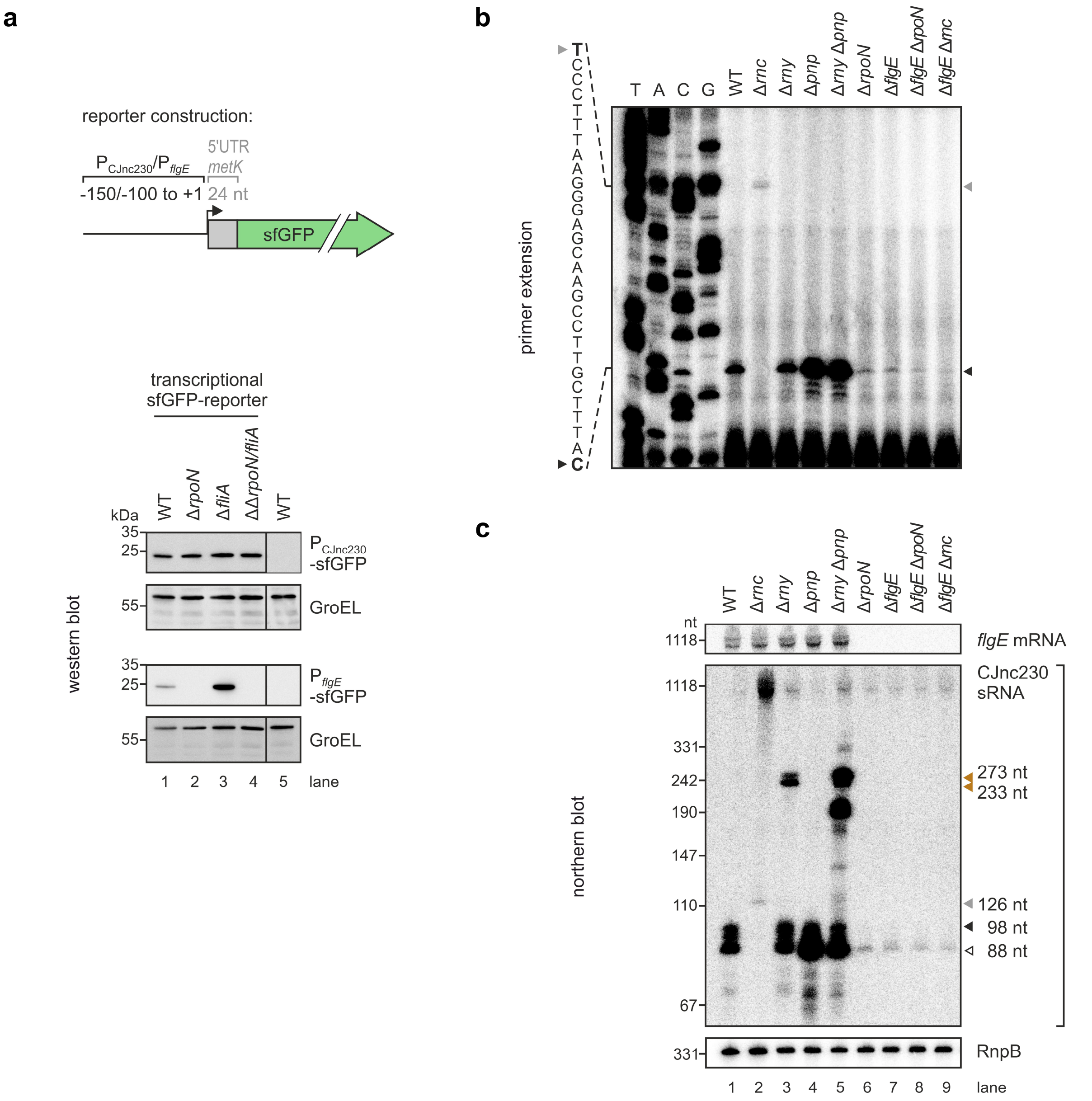

Supplementary Figure 5. A minor fraction of CJnc230 sRNA originates from an RpoD-dependent promoter. a, (*Upper*) *C. jejuni* transcriptional reporter construction. Either 100 nucleotides upstream of the *flgE* TSS[2] or 150 nucleotides upstream of the CJnc230 5' end/processing site (**Fig. 2b**) were fused to an unrelated ribosome binding site (RBS) (*metK*) and *sfgfp*. This construct was introduced together with a resistance cassette into the Cj0046 pseudogene locus. (*Lower*) Western blot analysis of $P_{CJnc230}$ and P_{flgE} transcriptional reporters in *C. jejuni* WT and sigma factor deletion strains grown to exponential phase. GroEL was detected for normalization. Representative images were cut between lanes 4 and 5. **b**, Primer extension analysis of total RNA from *C. jejuni* WT, ribonuclease deletion mutants, and Δ*rpoN*, Δ*flgE*, or Δ*flgE* mutants combined with deletion of *rpoN* or *rnc*, harvested at exponential phase. Lanes 1-5 are identical to main **Figure 2b**. Total RNA was annealed with the probe for CJnc230 used for northern blots (CSO-0537), binding in the middle of the sRNA. A sequencing ladder generated with this probe is partially indicated on the left. The gray triangle marks the alternative RpoD-dependent TSS (T residue in bold on the left) detected in Δ*rnc* bacteria (**Supplementary Fig. 4**), 28 nt upstream of the sRNA 5' end (C residue in bold on the left, black triangle) in WT. **c**, Northern

blot analysis of total RNA from *C. jejuni* WT, ribonuclease deletion mutants, and Δ*rpoN*, Δ*flgE*, or Δ*flgE* mutants combined with deletion of *rpoN* or *rnc*, harvested at exponential growth phase. Lanes 1-5 are identical to main **Figure 2a**. Lengths of prominent CJnc230 transcripts are indicated by colored triangles and were determined by term-seq and/or primer extension. Expression of CJnc230 sRNA was detected with CSO-0537, *flgE* mRNA with CSO-5136 (binding the *flgE* CDS), and RnpB RNA (CSO-0497) served as a loading control. Data in (**a-c**) are representative results of at least three independent experiments. Source data are provided as a Source Data File.

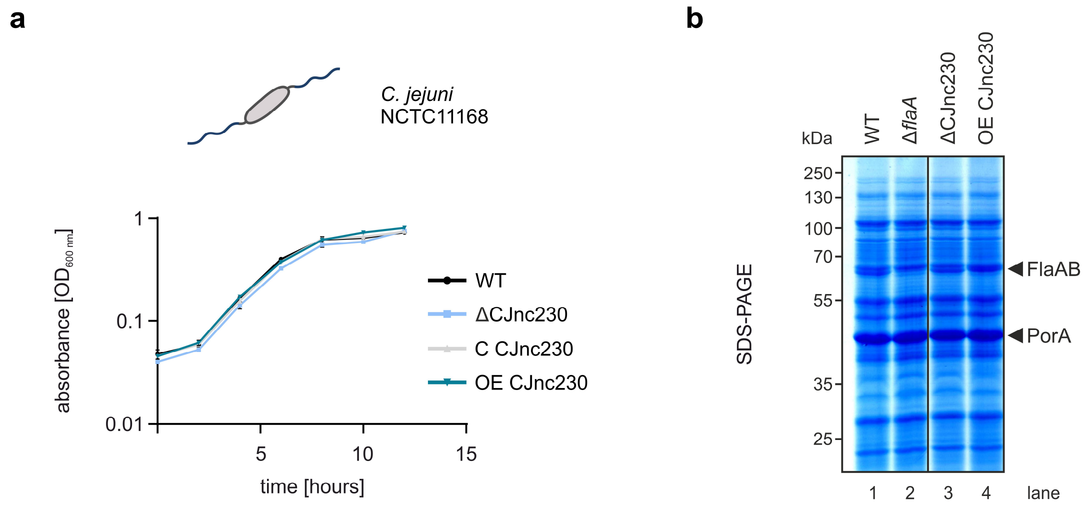

Supplementary Figure 6. Growth behavior and analysis of total protein patterns of *C. jejuni* CJnc230 mutant strains. a, Growth curve analysis of *C. jejuni* WT and CJnc230 deletion (Δ), complementation (C), and overexpression (OE) mutants. Strains were grown for 12 hours in Brucella broth (BB) medium and culture density ($OD_{600\ nm}$) was measured every 2 hours. The mean of two independent biological replicates ± standard deviation is shown. **b**, Total protein samples from *C. jejuni* WT and CJnc230 mutant strains (Δ and OE) harvested at exponential phase (t = 8 hrs). On the SDS-PAGE image, the flagellins (FlaA/B) and the non-regulated major outer membrane protein PorA (loading control) are indicated. Δ*flaA*: negative control. Image was cut between lanes 2 and 3. Data in (**a, b**) are representative results of at least two independent experiments. Source data are provided as a Source Data File.

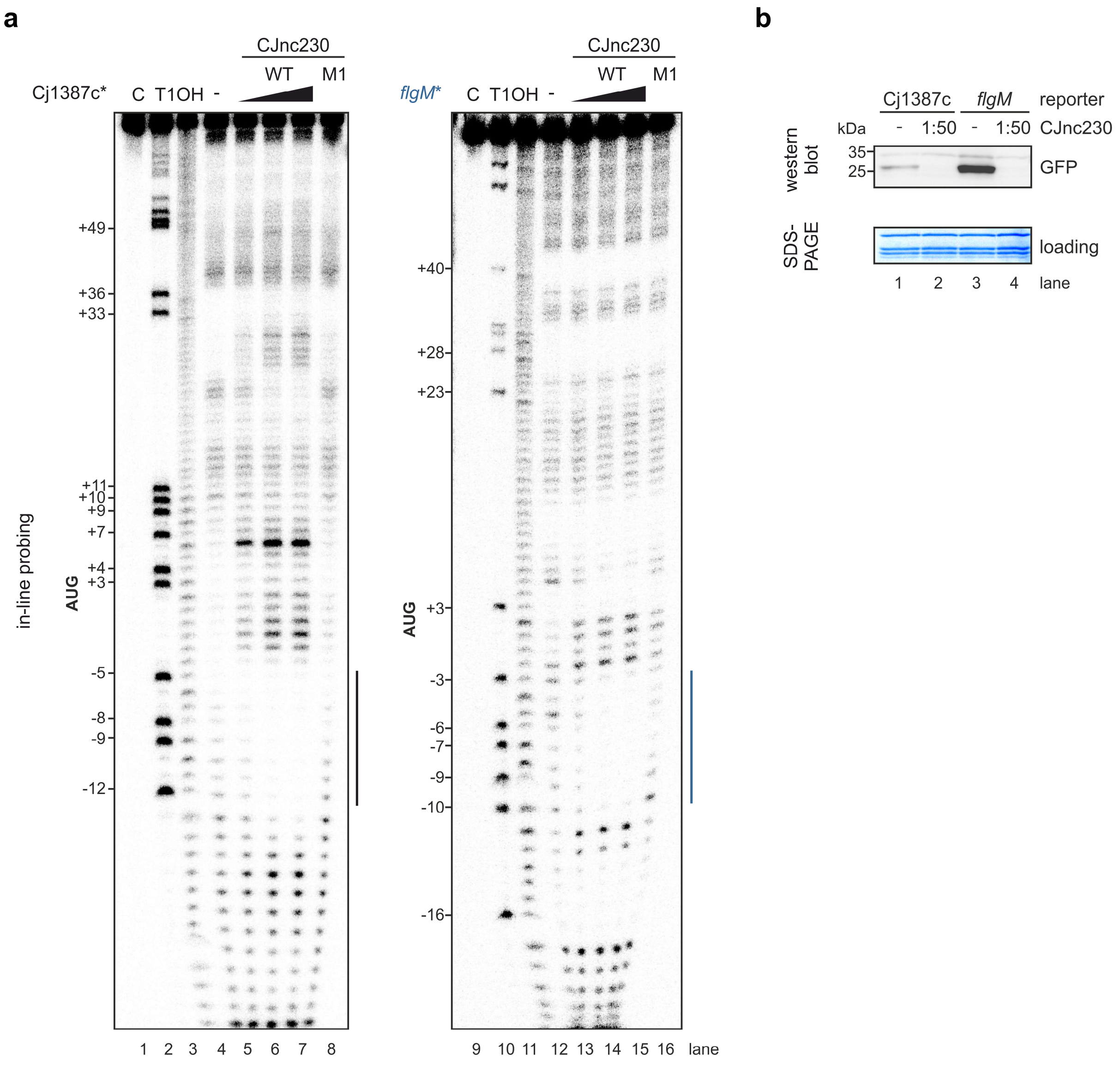

Supplementary Figure 7. *In-vitro* in-line probing and translational reporter assays confirm Cj1387c and *flgM* mRNAs as direct targets of CJnc230 repression. a, In-line probing of 0.2 pmol ^{32}P-5'-end-labeled (marked with *) Cj1387c and *flgM* mRNA leaders in the absence or presence of 0.02/0.2/2 pmol unlabeled CJnc230 WT sRNA or 2 pmol M1 mutant sRNA. Interaction sites with Cj1387c (black, left gel) and *flgM* (blue, right gel) 5'UTRs are indicated on the right. C - untreated control; T1 ladder - G residues (indicated on the left); OH - all positions (alkaline hydrolysis). **b**, *In-vitro* translation of Cj1387c- and *flgM*-sfGFP reporters (5'UTR and first 10 codons fused to *sfgfp*, 4 pmol) in an *E. coli* cell-free system -/+ CJnc230 (1:50 = 200 pmol) detected by western blot. PageBlue staining of the gel after blotting served as a loading control. Data in (**a, b**) are representative results of at least two independent experiments. Source data are provided as a Source Data File.

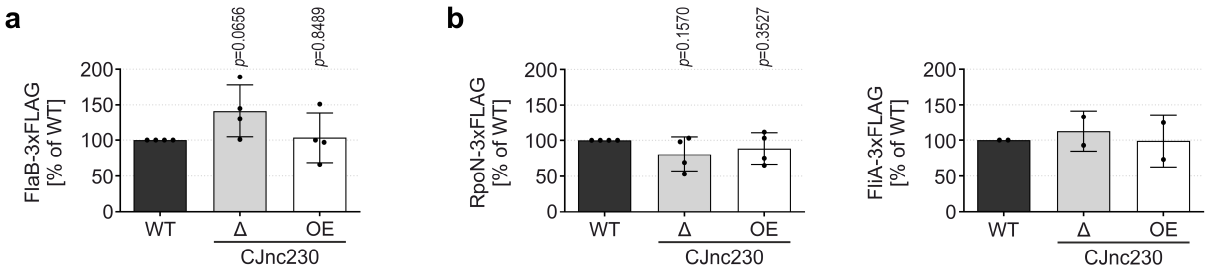

Supplementary Figure 8. CJnc230 does not affect levels of the minor flagellin FlaB or flagellar sigma factors. a,**b**, Protein levels of C-terminally FLAG-tagged FlaB (**a**) or RpoN (*left*) and FliA (*right*) (**b**) in *C. jejuni* grown to exponential phase measured by western blot. Bar graphs represent the mean of independent biological replicates (n = 4 for FlaB-3xFLAG; n = 4 for RpoN-3xFLAG; n = 2 for FliA-3xFLAG), error bars depict the standard deviation. Unpaired, two-tailed Student's *t*-test was used to compare the respective mutant to WT. Data in (**a, b**) are representative results of at least two independent experiments. Source data are provided as a Source Data File.

a **b**

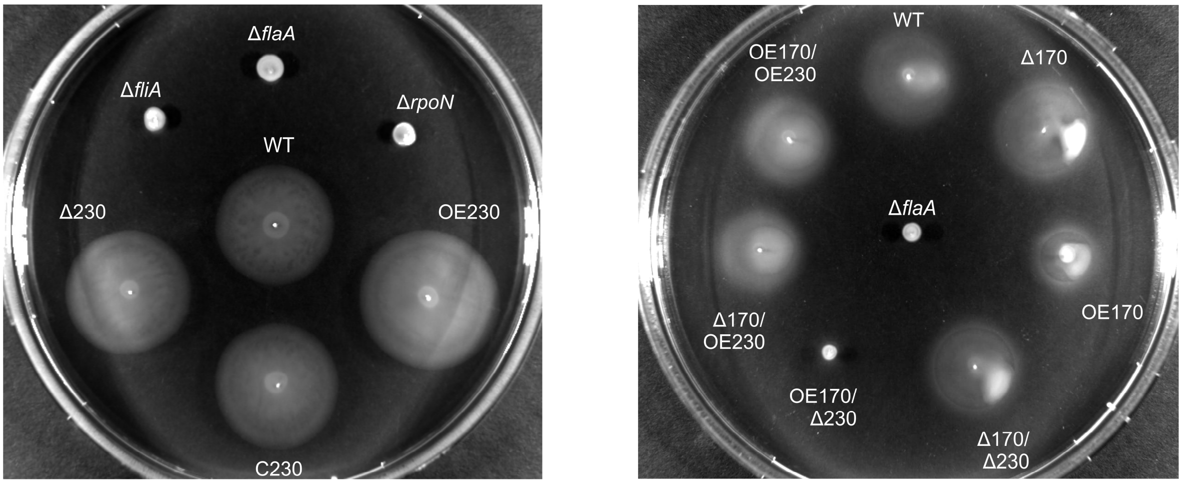

Supplementary Figure 9. Motility assays with sRNA single or double mutants reveal opposite phenotypes and motility regulation. a,**b**, Representative images of swimming motility assays of *C. jejuni* WT and CJnc230 mutant strains (Δ230: CJnc230 deletion, C230: CJnc230 complementation in *trans*, OE230: CJnc230 overexpression in *trans*) (**a**) or CJnc170 deletion (Δ170) or overexpression (OE170) mutants with or without deletion or overexpression of CJnc230 (**b**) in 0.4% soft agar BB plates imaged after 24 hrs of incubation. Δ*flaA*/Δ*fliA*/Δ*rpoN*: non-motile controls lacking major flagellin or flagellar sigma factors FliA and RpoN. Related to main **Figures 4b and 6a**. Data in (**a, b**) are representative results of at least six independent experiments. Source data are provided as a Source Data File.

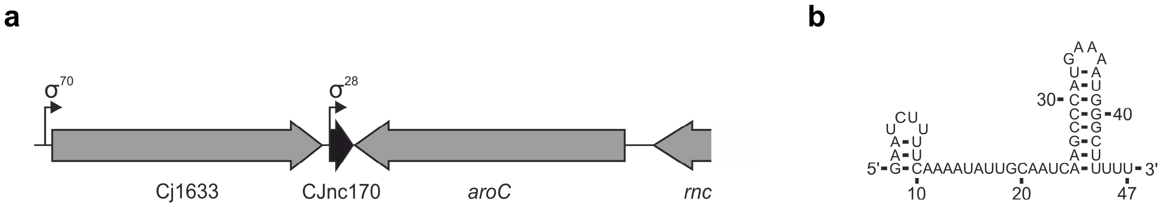

Supplementary Figure 10. CJnc170 sRNA is transcribed from a FliA-dependent promoter. **a**, Genomic location of CJnc170 sRNA in the intergenic region of Cj1633 (tRNA-uridine 2-sulfurtransferase) and Cj1634c (*aroC*, chorismate synthase) in *C. jejuni* NCTC11168[5]. The neighboring genes have no obvious relationship to motility. **b**, Secondary structure of the 47-nt long CJnc170 sRNA predicted by RNAfold[1].

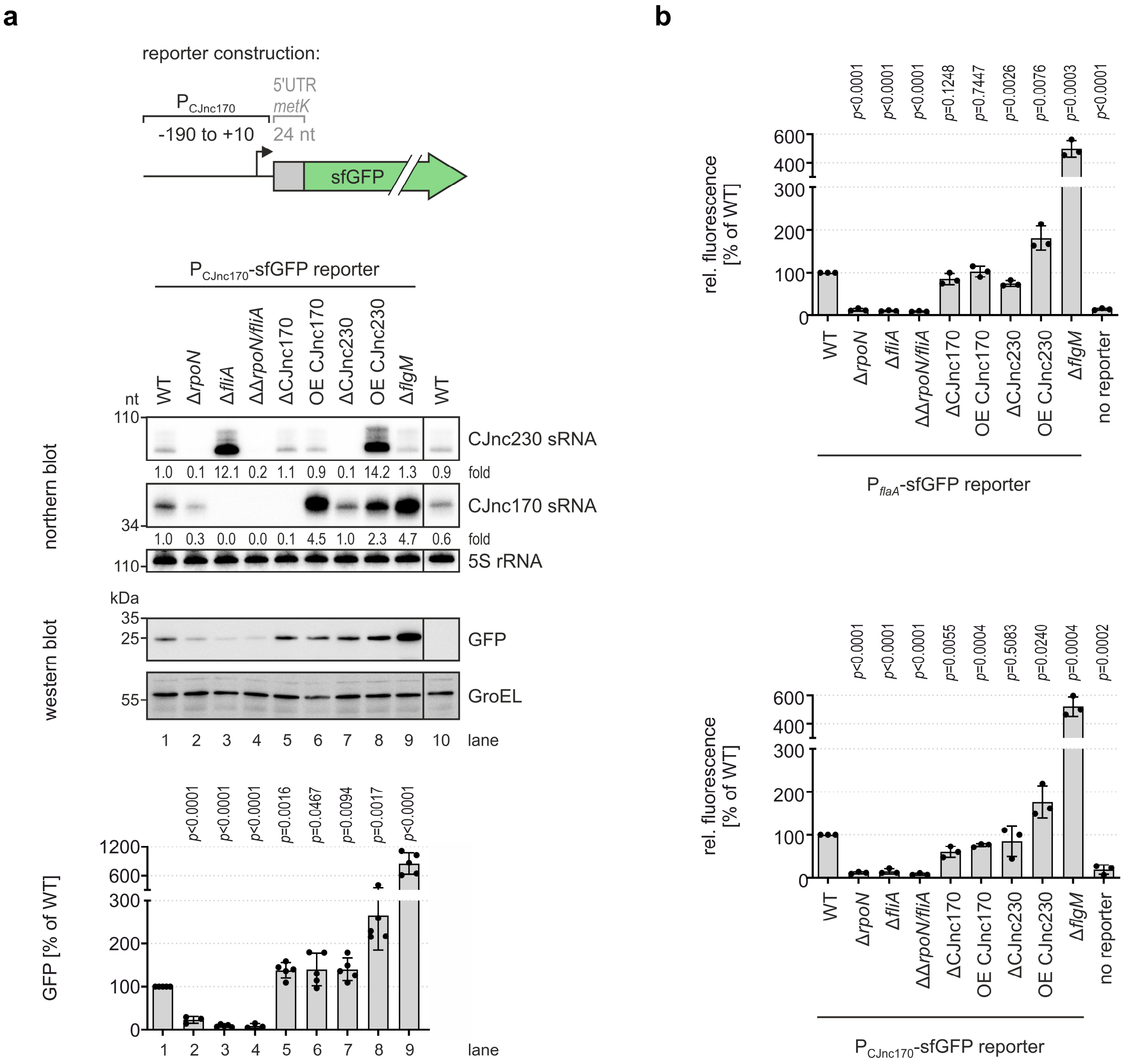

Supplementary Figure 11. Transcriptional reporter assays confirm CJnc230 activation of class III flagellar gene transcription. a, Northern and western blot analyses of *C. jejuni* WT or sigma factor, sRNA, and anti-sigma factor mutant strains with a transcriptional reporter of the $P_{CJnc170}$ promoter region fused to an unrelated RBS (*metK*) and *sfgfp*, harvested at exponential growth phase. Nucleotide positions with respect to the CJnc170 TSS[2,5] are indicated in the scheme on top. (*Middle panel*) Northern blot validation of CJnc230 (CSO-0537) and CJnc170 (CSO-0182) sRNA expression. 5S rRNA (CSO-0192) was used as a loading control. Fold changes of sRNA expression relative to the WT reporter and normalized to 5S rRNA are indicated. Images were cut between lanes 9 and 10. (*Lower panel*) Reporter expression in the respective mutants was measured by western blotting. GroEL was detected for normalization. One representative blot (cut between lanes 9 and 10) is shown and quantification of independent biological replicates (n = 5 for WT, Δ*fliA*, ΔCJnc170, OE CJnc170, ΔCJnc230, OE CJnc230, and Δ*flgM*; n = 3 for Δ*rpoN* and Δ*rpoN*/Δ*fliA*) is depicted in the bar graph below. Error bars represent the standard deviation. Unpaired, two-tailed Student's *t*-test was used to compare the respective mutant to the WT reporter background (lane 1). **b**, Flow cytometry analyses of P_{flaA} (*upper*) and $P_{CJnc170}$ (*lower*) transcriptional reporter expression at exponential growth phase. Bar graphs based on 100,000 counted cells per sample represent the mean of independent biological replicates (n = 3), error

bars depict the standard deviation. Unpaired, two-tailed Student's *t*-test was used to compare the respective mutant to the WT reporter background (WT). Data in (**a, b**) are representative results of at least three independent experiments. Source data are provided as a Source Data File.

a

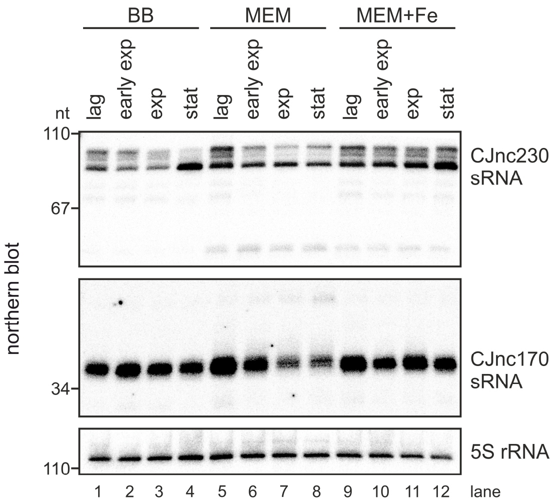

b

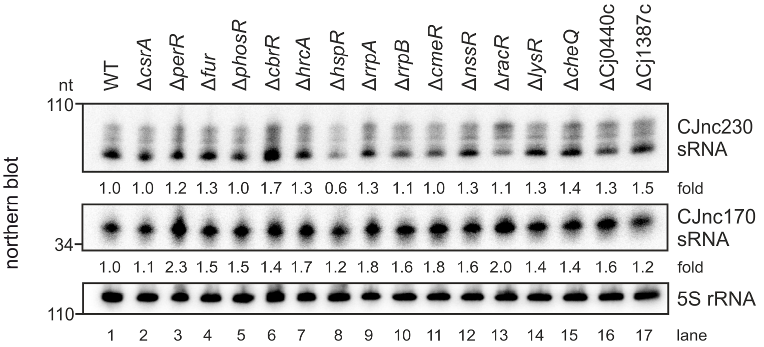

Supplementary Figure 12. Northern blot analyses of CJnc230 and CJnc170 expression. **a**, Total RNA of *C. jejuni* NCTC11168 WT grown in rich Brucella broth (BB) or minimal media (MEM) -/+ iron (Fe) was harvested at different growth phases: lag phase, $OD_{600\,nm}$ ~0.1 (lag); early exponential phase, $OD_{600\,nm}$ ~0.25 (early exp); exponential phase, $OD_{600\,nm}$ ~0.5 (exp); and stationary phase, $OD_{600\,nm}$ ~0.8 (stat). **b**, Total RNA from *C. jejuni* NCTC11168 WT and several transcriptional regulator mutant strains grown to exponential phase ($OD_{600\,nm}$ ~0.5). Fold changes of sRNA expression relative to WT and normalized to 5S rRNA are indicated. CJnc230 sRNA was detected with CSO-0537 and CJnc170 sRNA with CSO-0182. 5S rRNA (CSO-0192) served as loading control. Data in (**a, b**) are representative results of at least two independent experiments. Source data are provided as a Source Data File.

Supplementary References

1. Gruber, A. R., Lorenz, R., Bernhart, S. H., Neuböck, R. & Hofacker, I. L. The Vienna RNA websuite. *Nucleic Acids Res.* **36**, W70-4 (2008).
2. Porcelli, I., Reuter, M., Pearson, B. M., Wilhelm, T. & van Vliet, A. H. M. Parallel evolution of genome structure and transcriptional landscape in the Epsilonproteobacteria. *BMC Genomics* **14**, 616 (2013).
3. Sharma, C. M. *et al.* The primary transcriptome of the major human pathogen *Helicobacter pylori*. *Nature* **464**, 250–255 (2010).
4. Corpet, F. Multiple sequence alignment with hierarchical clustering. *Nucleic Acids Res.* **16**, 10881–10890 (1988).
5. Dugar, G. *et al.* High-resolution transcriptome maps reveal strain-specific regulatory features of multiple *Campylobacter jejuni* isolates. *PLoS Genet.* **9**, e1003495 (2013).
