# Supplementary material for: Interplay of two small RNAs fine-tunes hierarchical flagella gene expression in Campylobacter jejuni
Source: Nat Commun. 2024 Jun 19;15:5240. doi: 10.1038/s41467-024-48986-8 (PMC11187230; doi:10.1038/s41467-024-48986-8)
Supplement: Supplementary file 3 — Description of Additional Supplementary Files [file 41467_2024_48986_MOESM3_ESM.docx]

**Description of Additional Supplementary Files**

**Supplementary Dataset 1:**

Predicted targets of full-length CJnc230 (98 nt) in *C. jejuni* NCTC11168. The IntaRNA tool (Mann et al. 2017; PMID: 28472523) was used to predict interaction of full-length CJnc230 (98 nt) with *C. jejuni* NCTC11168 (NC_002163.1). A *p*-value cutoff of ≤ 0.05 was used, which includes the validated targets *flgM* (Cj1464) and Cj1387c on top. Overlapping predicted targets to 3'-truncated CJnc230 (Supplementary Dataset 2) are indicated in the last column.

**Supplementary Dataset 2:**

Predicted targets of 3'-truncated CJnc230 (88 nt) in *C. jejuni* NCTC11168. The IntaRNA tool (Mann et al. 2017; PMID: 28472523) was used to predict interaction of 3'-truncated CJnc230 (88 nt) with *C. jejuni* NCTC11168 (NC_002163.1). A *p*-value cutoff of ≤ 0.05 was used, which includes the validated targets *flgM* (Cj1464) and Cj1387c.

**Supplementary Dataset 3:**

Filament lengths of *C. jejuni* NCTC11168 CJnc230 mutants. The length of flagellar filaments was quantified from raw electron micrographs using the ridge detection plug-in of ImageJ. Mean filament lengths [nm] with standard deviation (SD) are indicated at the bottom.

**Supplementary Dataset 4:**

Filament lengths of *C. jejuni* NCTC11168 sRNA double mutant strains. The length of flagellar filaments was quantified from raw electron micrographs using the ridge detection plug-in of ImageJ. Mean filament lengths [nm] with standard deviation (SD) are indicated at the bottom.

**Supplementary Dataset 5:**

Differentially expressed genes in *C. jejuni* NCTC11168 Δ*rny* in comparison to the wildtype. DESeq2 analysis (Love et al. 2014; PMID: 25516281) was performed to quantify gene expression changes in bacteria lacking RNase Y in comparison to the WT. Genes with a log2 fold-change (log2FC) ≥ |1| and an adjusted (Benjamini-Hochberg corrected) *p*‑value (padj) ≤ 0.05 were considered as differentially expressed. Several RNA types were distinguished: tRNA, sRNA, 5'UTR, and coding sequence (CDS).

**Supplementary Dataset 6:**

List of bacterial strains used in this study. Abbreviations for antibiotic resistances are as follows: GmR - *aac(3)-IV* (gentamicin, encoding an aminoglycoside acetyltransferase); KanR - *aphA-3* (kanamycin, aminoglycoside phosphotransferase); CmR - *cat* (chloramphenicol acetyltransferase); HygR - *aph(7″)* (hygromycin B, aminoglycoside phosphotransferase); TetR - *tetO* (tetracycline, encoding a ribosomal protection/resistance protein). WT: wildtype; Δ: deletion; C: complementation in *trans*; OE: overexpression in *trans*; F: FLAG; P: promoter; 5’UTR: 5’ untranslated region. For *E. coli* strains harboring cloned plasmids, see Supplementary Dataset 7.

**Supplementary Dataset 7:**

List of plasmids used in this study. AmpR - ampicillin resistance (*bla*); GmR - gentamicin resistance (*aac(3)-IV*); KanR - kanamycin resistance (*aphA-3*); CmR - chloramphenicol resistance (*cat*); HygR - hygromycin B resistance (*aph(7″)*). P: promoter; 5'UTR: 5' untranslated region; CDS: coding sequence.

**Supplementary Dataset 8:** Deoxyoligonucleotides used for cloning, northern hybridisation, and T7 transcription template generation. P denotes a 5' monophosphate, mutations are highlighted in red. NB: northern blot; SDM: site-directed mutagenesis; RT: reverse transcription.

**Supplementary Dataset 9:** Translational and transcriptional sfGFP reporters used in this study. Putative promoter motifs and transcriptional start sites (TSSs) are marked in red and orange, respectively, and ribosome binding sites (RBSs) and start codons are underlined. Genomic regions surrounding the promoter motifs included for reporter construction are shown in gray and the *sfgfp* sequence is highlighted in green. Translational reporters were generated by fusing the 5' untranslated region (UTR) and the first ten codons of the respective gene to the second codon of *sfgfp*.

**Supplementary Dataset 10:** T7-transcripts used in this study. Ribosome binding sites and start codons are underlined. Mutations are highlighted in red and the *sfgfp* sequence starting at the second codon in green.
