# Supplementary material for: Interplay of two small RNAs fine-tunes hierarchical flagella gene expression in Campylobacter jejuni
Source: Nat Commun. 2024 Jun 19;15:5240. doi: 10.1038/s41467-024-48986-8 (PMC11187230; doi:10.1038/s41467-024-48986-8)
Supplement: Supplementary file 14 — Reporting Summary [file 41467_2024_48986_MOESM14_ESM.pdf]

nature portfolio

Corresponding author(s): Cynthia M. Sharma

Last updated by author(s): Apr 25, 2024

Reporting Summary

Nature Portfolio wishes to improve the reproducibility of the work that we publish. This form provides structure for consistency and transparency in reporting. For further information on Nature Portfolio policies, see our Editorial Policies and the Editorial Policy Checklist.

Statistics

For all statistical analyses, confirm that the following items are present in the figure legend, table legend, main text, or Methods section.

n/a	Confirmed	
☐	☒	The exact sample size (*n*) for each experimental group/condition, given as a discrete number and unit of measurement
☐	☒	A statement on whether measurements were taken from distinct samples or whether the same sample was measured repeatedly
☐	☒	The statistical test(s) used AND whether they are one- or two-sided<br>*Only common tests should be described solely by name; describe more complex techniques in the Methods section.*
☒	☐	A description of all covariates tested
☒	☐	A description of any assumptions or corrections, such as tests of normality and adjustment for multiple comparisons
☐	☒	A full description of the statistical parameters including central tendency (e.g. means) or other basic estimates (e.g. regression coefficient) AND variation (e.g. standard deviation) or associated estimates of uncertainty (e.g. confidence intervals)
☐	☒	For null hypothesis testing, the test statistic (e.g. *F*, *t*, *r*) with confidence intervals, effect sizes, degrees of freedom and *P* value noted<br>*Give P values as exact values whenever suitable.*
☒	☐	For Bayesian analysis, information on the choice of priors and Markov chain Monte Carlo settings
☒	☐	For hierarchical and complex designs, identification of the appropriate level for tests and full reporting of outcomes
☒	☐	Estimates of effect sizes (e.g. Cohen's *d*, Pearson's *r*), indicating how they were calculated

Our web collection on statistics for biologists contains articles on many of the points above.

Software and code

Policy information about availability of computer code

Data collection	blastn (PMID: 2231712), GLASSgo (PMID: 29719549), and KEGG (https://www.genome.jp/kegg/) were used for identification of sRNA and flgE homologs. Alignments were performed using MultAlin (PMID: 2849754). RNA structure prediction and folding was done using RNAfold (PMID: 18424795) and VARNA (PMID: 19398448). RNA-RNA interaction predictions were computed genome-wide with IntaRNA (PMID: 28472523) version 3.2.0 (linking Vienna RNA package 2.4.14) using default parameters, except for seed size of 6 nt.<br><br>For flow cytometry, a BD Accuri C6 plus instrument (version 1.0.23.1, build 20151211.23.1) was used. Transmission electron microscopy was performed on a JEOL-2100 microscope. Fluorescently-labelled bacteria were imaged with a Leica TCS SP5 II laser scanning confocal microscope (Leica Microsystems) with LAS AF software (version 2.7.3.9723).

Data analysis	Western blot images and images of motility assays were acquired with an ImageQuant LAS 4000 device (GE; version 1.3, build 1.3.0.134). Images from northern blot, primer extension, and in-line probing assays were taken on a Typhoon FLA-7000 series PhosphorImager (GE; version 1.3, build 1.3.0.105). Densitometry analyses were conducted using AIDA image analysis software (v5.0, build 1182, Raytest, Germany) and images were edited with ImageJ (NIH, USA; v 1.53j). Pictures of protein gels were taken on an Amersham ImageQuant 800 instrument (GE; 06340134, version 1.2.0). Statistical tests were performed with GraphPad Prism (GraphPad Software, CA, USA; v 9.2.0).<br><br>Flagellar length measurements of electron micrographs were performed with ImageJ software (NIH, USA; v 1.53j) and the ridge detection plug-in applying the following settings: line width: 12; high contrast: 150; low contrast: 50; sigma: 3.96; lower threshold: 0.34; upper threshold: 0.85; minimum line length: 100; maximum line length: 6,000. Statistical tests were performed with GraphPad Prism (GraphPad Software, CA, USA; v 9.2.0).

For manuscripts utilizing custom algorithms or software that are central to the research but not yet described in published literature, software must be made available to editors and reviewers. We strongly encourage code deposition in a community repository (e.g. GitHub). See the Nature Portfolio guidelines for submitting code & software for further information.

Data

Policy information about availability of data

All manuscripts must include a data availability statement. This statement should provide the following information, where applicable:

- Accession codes, unique identifiers, or web links for publicly available datasets
- A description of any restrictions on data availability
- For clinical datasets or third party data, please ensure that the statement adheres to our policy

The total RNA-seq, dRNA-seq, and term-seq datasets have been deposited at the NCBI Gene Expression Omnibus (PMID: 11752295; https://www.ncbi.nlm.nih.gov/geo/) under the accessions GSE230835 [https://www.ncbi.nlm.nih.gov/geo/query/acc.cgi?acc=GSE230835], GSE230836 [https://www.ncbi.nlm.nih.gov/geo/query/acc.cgi?acc=GSE230836], and GSE230837 [https://www.ncbi.nlm.nih.gov/geo/query/acc.cgi?acc=GSE230837], respectively. The reference genome sequence NC_002163.1 (ASM908v1) and annotation was recovered from NCBI (2014-03-20). The sRNA and 5'UTR annotation was generated from published differential RNA-seq data (Dugar et al. 2013, PMID: 23696746) retrieved from GEO (accession GSE38883, [https://www.ncbi.nlm.nih.gov/geo/query/acc.cgi?acc=GSE38883]). All other data supporting the findings of this study are available within the article and its supplementary files. Source data are provided with this paper.

Research involving human participants, their data, or biological material

Policy information about studies with human participants or human data. See also policy information about sex, gender (identity/presentation), and sexual orientation and race, ethnicity and racism.

Reporting on sex and gender	n/a
Reporting on race, ethnicity, or other socially relevant groupings	n/a
Population characteristics	n/a
Recruitment	n/a
Ethics oversight	n/a

Note that full information on the approval of the study protocol must also be provided in the manuscript.

Field-specific reporting

Please select the one below that is the best fit for your research. If you are not sure, read the appropriate sections before making your selection.

- [x] Life sciences
- [ ] Behavioural & social sciences
- [ ] Ecological, evolutionary & environmental sciences

For a reference copy of the document with all sections, see nature.com/documents/nr-reporting-summary-flat.pdf

Life sciences study design

All studies must disclose on these points even when the disclosure is negative.

Sample size	All northern blots, western blots, RT-PCR experiments, flow cytometry experiments, and motility assays shown are representative of at least 2 independent biological replicates. RNA-seq and term-seq datasets were generated for sets of three or two independent biological samples (cultures), respectively. dRNA-seq was not performed in replicates as no global analysis was performed and only one gene locus is shown in the Supplementary Information to confirm results obtained in complementary experiments. Sample sizes were selected based on prior experience with similar experimental setups to determine statistical significance as previously published in studies from our lab (PMIDs: 23696746, 27229370, 32069333, 34290242, 34843430, 34818434).
Data exclusions	No data was excluded.

Replication	All experiments were reproduced at least two times and replications were successful. dRNA-seq was performed once to confirm results obtained in complementary experiments within this study.
Randomization	Randomization was not relevant for the study as corresponding samples were prepared together.
Blinding	Blinding was not relevant as none of the readouts was subjective.

Reporting for specific materials, systems and methods

We require information from authors about some types of materials, experimental systems and methods used in many studies. Here, indicate whether each material, system or method listed is relevant to your study. If you are not sure if a list item applies to your research, read the appropriate section before selecting a response.

Materials & experimental systems

n/a	Involved in the study
☐	☒ Antibodies
☒	☐ Eukaryotic cell lines
☒	☐ Palaeontology and archaeology
☒	☐ Animals and other organisms
☒	☐ Clinical data
☒	☐ Dual use research of concern
☒	☐ Plants

Methods

n/a	Involved in the study
☒	☐ ChIP-seq
☐	☒ Flow cytometry
☒	☐ MRI-based neuroimaging

Antibodies

Antibodies used	1) primary antibody: monoclonal mouse anti-FLAG; 1:1,000; Sigma-Aldrich; #F1804-1MG; RRID:AB_262044<br>2) primary antibody: monoclonal mouse anti-GFP; 1:1,000; Roche #11814460001; RRID:AB_390913<br>3) secondary antibody: polyclonal sheep anti-mouse IgG horseradish peroxidase (HRP) conjugate; 1:10,000; GE Healthcare; #RPN4201<br>4) primary antibody: antibody specific for GroEL (rabbit polyclonal); 1:10,000; Sigma-Aldrich; #G6532-5ML; RRID:AB_259939<br>5) secondary antibody: polyclonal goat anti-rabbit IgG; 1:10,000; GE Healthcare; #RPN4301; RRID:AB_2650489
Validation	All primary antibodies have been validated by the manufacturers and have been previously used for the same applications (GroEL, FLAG, GFP; Pernitzsch et al. 2021, PMID: 34290242). On each western blot (except for in-vitro translation assays) an untagged wild-type control was included to distinguish specific from unspecific detection.

Flow Cytometry

Plots

Confirm that:

☐ The axis labels state the marker and fluorochrome used (e.g. CD4-FITC).

☒ The axis scales are clearly visible. Include numbers along axes only for bottom left plot of group (a 'group' is an analysis of identical markers).

☐ All plots are contour plots with outliers or pseudocolor plots.

☒ A numerical value for number of cells or percentage (with statistics) is provided.

Methodology

Sample preparation	For single cell analysis of sfGFP reporters in C. jejuni, bacteria grown to mid-log phase (OD600 nm 0.4-0.5) were collected by centrifugation at 7,500 x g and 4°C for 5 min. Pellets were resuspended in 500 µl 4% paraformaldehyde/PBS and fixed overnight at 4°C. After two washes with PBS, cells were resuspended in PBS and stored at 4°C.
Instrument	BD Accuri C6 plus (R660517590225)
Software	For flow cytometry, a BD Accuri C6 plus instrument (software version 1.0.23.1, build 20151211.23.1) was used. Analysis was done using FlowJo software (FlowJo, OR, USA; v10) and statistical tests were performed with GraphPad Prism (GraphPad Software, CA, USA; v 9.2.0).
Cell population abundance	Bacterial cells were resuspended in PBS and 100,000 events per sample were measured. sfGFP expression of C. jejuni strains was quantified without specific gating and sorting for three independent biological replicates.

Gating strategy	A lower cutoff of 2,000 was set for the forward scatter (FSC-H). No specific gating strategy was applied and fluorescence of all collected cells considered for subsequent analysis.

☐ Tick this box to confirm that a figure exemplifying the gating strategy is provided in the Supplementary Information.
